# Strengthening the biokinetics workforce for improved services: A human resources for health review from 2000 to 2020

**DOI:** 10.17159/2078-516X/2023/v35i1a14184

**Published:** 2023-04-05

**Authors:** R Tiwari, HW Grobbelaar, C Vermaak, U Chikte

**Affiliations:** 1 Division of Health Systems and Public Health, Department of Global Health, Faculty of Medicine and Health Sciences, Stellenbosch University, Tygerberg, South Africa; 2 Division of Sport Science, Department of Exercise, Sport and Lifestyle Medicine, Faculty of Medicine and Health Sciences, Stellenbosch University, Stellenbosch, South Africa; 3 Division of Movement Science and Exercise Therapy, Department of Exercise, Sport and Lifestyle Medicine, Faculty of Medicine and Health Sciences, Stellenbosch University, Stellenbosch, South Africa

**Keywords:** clinical exercise therapy, HPCSA registration, training

## Abstract

**Background:**

Biokinetics is a South African (SA) health profession within the private health care sector. Biokineticists register with the Health Professions Council of SA (HPCSA).

**Objectives:**

To describe the demographic trends of HPCSA registered biokineticists from 2000 to 2020 to understand the supply and status of human resources for health within the profession.

**Methods:**

The following data were collected and analysed: i) health personnel category, ii) geographical location, iii) age, iv) sex, and v) population category.

**Results:**

The number of HPCSA registered biokineticists grew from 136 in 2000, to 1831 in January 2020 (67.8% women, 32.2% men). There was a sharp decline in numbers after the age of 45 years. The Western Cape (5.8) and Gauteng (5.1) provinces had the most biokineticists per 100 000 of the population, whilst smaller provinces like Kwazulu-Natal (1.6), Mpumalanga (1.6), North-West (1.6) and Limpopo (0.9) lagged. The demographic profile of registered Biokineticists changed steadily from 2000 to 2020. Registered biokineticists classified as White decreased from 91.6% to 80.4%, whilst substantial increases were observed among Black (5.0% to 8.3%), Coloured (0.02% to 5.3%) and Indian/Asian (0.02% to 6.0%) biokineticists. Thirteen tertiary institutions offered Biokinetics programmes in 2022. Seven offered the 3+1-year (Honours) programme and six have migrated to a 4-year professional degree.

**Conclusion:**

The profession is well established, growing, and dominated by women. The demographic profile has transformed steadily; however, the need to transform the profession remains strong. Strengthening investments aimed at the employment of biokineticists in the public health sector may serve as a key turning point for healthcare workforce planning.

Stellenbosch University was the first South African university to offer a programme in Physical Education.^[1]^ Biokinetics developed from the South African Physical Education Programme that can be traced to the 1930s.^[2]^ It is a South African health profession that functions predominantly in the private healthcare sector. Biokineticists register with the Health Professions Council of South Africa (HPCSA), which regulates the profession.^[3]^ The Biokinetics Association of South Africa (BASA) is the representative body, with a vision for the profession ‘to be a recognised leader and vital collaborator within the health sector’.^[4]^ Biokinetics originated in South Africa four decades ago when the first scope of practice was published in the South African Government Gazette in 1983^[2]^ and it has expanded internationally. Biokineticists in the United Kingdom are currently seeking registration as healthcare professionals,^[5]^ whilst the profession signed a memorandum of understanding with the professional organisation Exercise and Sport Science Australia.^[6]^

Biokineticists form part of multidisciplinary teams alongside medical doctors, physiotherapists, dieticians, psychologists, etc.^[3]^ They operate within the pathogenic (illness care and prevention), and fortogenic (health promotion) paradigms.^[2]^ The word ‘Biokinetics’ is derived from ‘Bio’ meaning life and ‘Kinesis’ meaning movement.^[3]^ These exercise therapy professionals use exercise and physical activity to treat pathologies, focussing on injury rehabilitation, promotion of health and quality of life, and the prevention and management of chronic diseases of lifestyle (CDL), as well as secondary and associated conditions.^[7]^ Biokineticists use physical activity and personalised exercise prescription as a therapeutic modality for final phase rehabilitation and injury management, prevention and management of chronic illnesses, lifestyle diseases and disabilities.^[2,8]^ Physiotherapists assess, treat and manage a variety of injuries, ailments and movement disorders to restore normal functioning, minimise pain and dysfunction through exercise and hands-on physical therapy, i.e. mobilisation, manipulation and massage.^[9]^

Biokineticists have an important role to play in view of the estimation that more than a billion individuals (15% of the world population) live with functional disabilities because of global increases in the burden of non-communicable diseases (NCDs) and musculoskeletal (MSK) disorders.^[10]^ About 74% of the total number of years lived with disability (YLD, with one YLD equalling one full year of healthy life lost because of disability or ill-health) are because of health conditions that cause functional restrictions that could benefit from exercise rehabilitation.^[11]^ Exercise rehabilitation benefits the individual and society at large by enhancing one’s independence, the ability to return to work, and participation in other social roles.

It also reduces the costs for care and support, as well as the length of hospitalisation and re-admission rates.^[12,13]^ Biokineticists are critical in meeting the current burden of disease by contributing to the prevention, promotion, treatment and support of individuals with functional capacity deficits and disabilities.^[3,14]^ They promote active lifestyles among individuals with NCDs through exercise, reducing the effects of sedentary behaviour and enhancing the ability to perform activities of daily living.^[3,14]^

South Africa as a developing country faces a quadruple burden of disease. The prevention and treatment of NCDs is already marginalised because of the high prevalence of communicable diseases. The secondary and associated conditions seen with communicable diseases (e.g. human immunodeficiency virus (HIV) / acquired immunodeficiency syndrome (AIDS) and tuberculosis), maternal and child mortality, NCDs (e.g. hypertension and cardiovascular diseases, diabetes, cancer, mental illnesses and chronic lung diseases, such as asthma), as well as injury and trauma further burden the under-resourced health sector.^[15]^ The number of YLDs is rising because of the decrease in mortality rates of South Africans with such conditions, as well as the rising ageing population.^[16]^ The increase in functional disability places a greater demand on physical exercise rehabilitation to restore functional ability and quality of life for affected individuals.^[16]^ Unfortunately, only 26% of people living in Southern Africa receive the exercise rehabilitation they need.^[11]^

On the health workforce front, South Africa needs to plan, support and upscale the supply pipeline of biokineticists to meet the health needs of society.^[3]^ This study aims to describe the demographic trends of biokineticists registered with the HPCSA from 2000 to 2020 as the first step towards understanding the supply and status of human resources for the health of biokineticists in South Africa. This study is inspired by a national need to review the biokineticist workforce to aid in the provision of a more robust and evidence-informed priority setting for health promotion, maintenance of physical abilities and final phase exercise rehabilitation.

## Methods

Ethical approval and a waiver of informed consent for this retrospective study was obtained from the Stellenbosch University Health Research Ethics Committee (HREC No: X21/05/009).

The study was a retrospective record-based review of the HPCSA database from 2000 until 2020. A similar approach was adopted to that of earlier studies.^[17,18]^ Relevant data were recorded using a data collection sheet with the following variables: (i) category of health personnel (Professional Board of Physiotherapy, Podiatry and Biokinetics), (ii) geographical location, (iii) age, (iv) sex, and (v) population category. The term ‘population’ was used in line with the definitions in the Population Registration Act (Act No. 30 of 1950), which previously classified South African citizens into four major population categories: White, Coloured, Indian/Asian and Black.^[19]^ Although the legislation was repealed in 1991, these population categories are still used in reporting in sectors such as the Department of Higher Education and the Department of Health. Racial data are still important in monitoring the redress in the education and training of health professionals who were previously denied access to such training in terms of the apartheid legislation.

The dataset was accessed, collated, and analysed by the leading author and the team members crosschecked the accuracy. Data were entered into a Microsoft Excel 2016 spreadsheet and analysed using the Statistical Package for the Social Sciences (SPSS version 22.0). Frequency distributions, cross tabulations and graphical representations were used as descriptive statistical methods. Anonymity and confidentiality of all HPCSA registered biokineticists were ensured because the data accessed from the HPCSA and presented in this paper was kept de-identified.

## Results

A total of 136 biokineticists were registered with the HPCSA in January 2000, and the number increased to 1831 in 2020, of which 19 were foreign nationals. The geographical distribution, age breakdown, demographic factors (sex and population group) for the 2020 data follows.

### Geographical (provincial) distribution

**Fig. 1:**
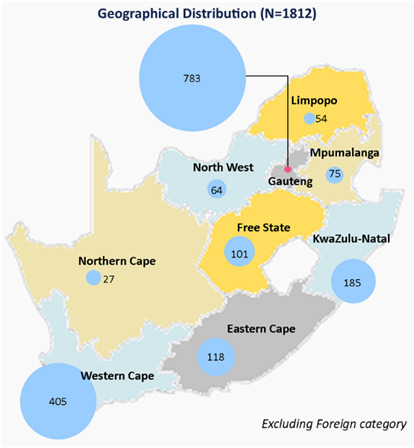
Geographical distribution of HPCSA registered biokineticists in South Africa (January 2020

In 2020, most biokineticists were in Gauteng (43.2%), the Western Cape (22.4%) and KwaZulu-Natal (10.2%). The Northern Cape (1.5%), Limpopo (3.0%) and the North-West province (3.5%) had the lowest numbers. The availability of biokineticists per 100 000 of the population was the highest in the Western Cape at 5.8, followed by Gauteng at 5.1 and the Free State at 3.4. The lowest availability was in the Limpopo province at 0.9 biokineticists per 100 000 of the population, with KwaZulu-Natal, Mpumalanga and the North-West province lagging with 1.6 each (see Figure 1 and Table 1).

### Age distribution

**Fig. 2:**
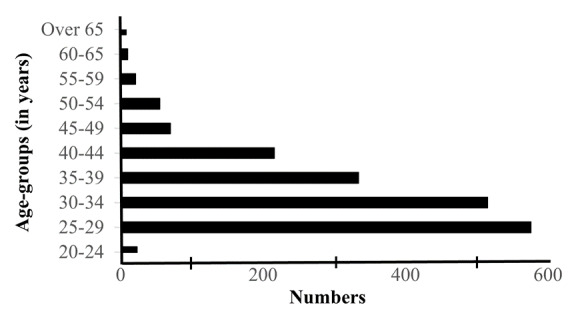
Age distribution of HPCSA registered biokineticists in South Africa (January 2020)

In 2020, most registered biokineticists were under the age of 40 years (79.2%). There was a sharp decline in the number of registered biokineticists after the age of 45 years (see Figure 2).

### Demographic profile of registered biokineticists (sex and population group)

**Fig. 3:**
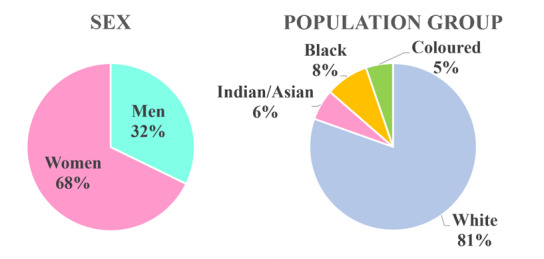
Sex and population group distribution of HPCSA registered biokineticists in South Africa (January 2020)

Figure 3 depicts the 2020 data for sex and population groups of registered biokineticists. In the workforce, 67.8% of the biokineticists were women (n = 1241) compared to 32.2% men (n = 590). The change in the population group data from 2000 to 2020 is shown in Figure 5.

### Growth in the number of biokineticists in South Africa

**Fig. 4:**
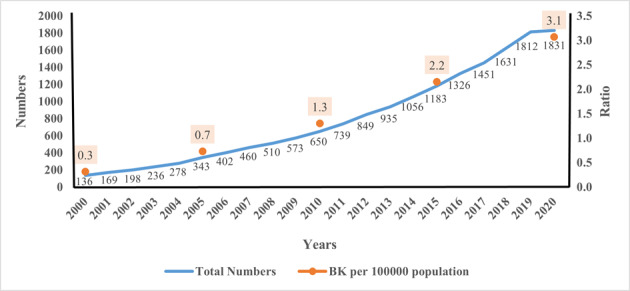
HPCSA registered biokineticists and population ratios from 2000 to 2020 in South Africa

Over the past decade (2010 to 2020), the number of registered biokineticists has almost tripled. There has been an overall increase of 1246.32% over the last 20 years. The ratio of biokineticists per 100 000 of the population has also increased from 0.3 in 2000 to 3.1 in 2020 (see Figure 4). South Africa had a population of 43054000 in 2000, which grew steadily to 59622351 in 2020.

### Population group trends from 2000 to 2020 in South Africa

**Fig. 5:**
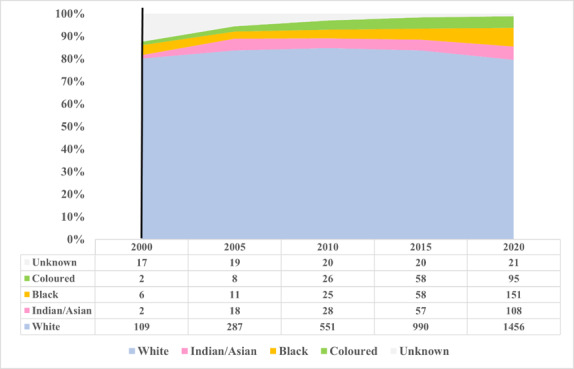
Five-yearly population group trends of HPCSA registered biokineticists (2000–2020)

Figure 5 depicts changes in the population group characteristics in five-year intervals (2000 to 2020). In 2000, 91.6% of the registered biokineticists were White, 5.0% Black, and 0.02% Coloured and Indian/Asian, respectively. In 2010, the biokineticists who were identified as White reduced to 87.5%, while Indians/Asians increased to 4.4%, Coloured to 4.1% and Black to 4.0%. This trend continued so that by 2020 the biokineticists who were identified as White reduced to 80.4%, whilst Indian/Asian, Black, and Coloured biokineticists increased to 6.0%, 8.3% and 5.3%, respectively.

### Demographic trends by age, population group and sex

**Fig. 6:**
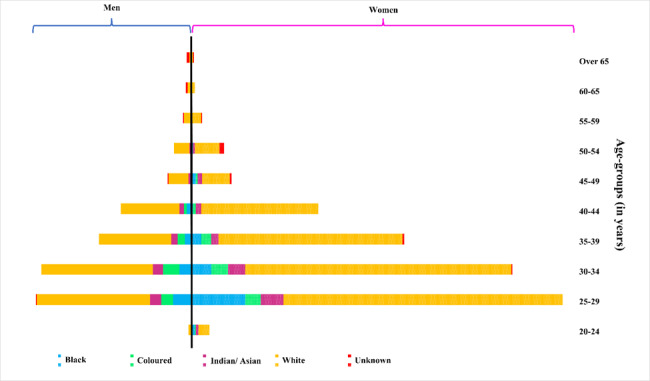
Breakdown of HPCSA registered biokineticists by age, sex, and population group (2020)

Figure 6 tracked the demographic variables by age, population group, and sex from 2000 to 2020. It showed that in 2020, women from the population group classified as White dominated the biokinetics workforce across all age categories except for the 55- to 65-year-olds. The number of biokineticists decreased with age across all population groups, with the decrease noticeably higher in the population classified as White. The largest proportional growth between 2015 and 2020 was noted among the Black biokineticists for both men and women.

### Training and education

Table 2 lists the 13 tertiary institutions accredited to offer biokinetics programmes in 2022 that enables registration with the HPCSA. The University of KwaZulu-Natal will not offer the programme after 2022. Of the 12 institutions offering programmes in 2023, four are based in the Gauteng province (33.3%), three in the Western Cape (25.0%), and one in each of the Eastern Cape, Free State, Kwazulu-Natal, Limpopo, and North-West provinces (8.3% each). Mpumalanga and the Northern Cape do not offer biokinetics programmes. Two new universities were set up in these provinces in 2014, but they have restricted academic offerings. The table also indicates the institutions that offer Master’s and PhD programmes. Students in these research programmes could focus on biokinetics-related topics, but the ensuing qualifications are not needed for registration, neither does it enable HPCSA registration.

## Discussion

Biokineticists mainly operate in the private healthcare sector, and the geographical distribution closely resembles the distribution of the capital of each province. The number of tertiary institutions that offer biokinetics programmes also influences the proportion of registered biokineticists per province (e.g. Gauteng and Western Cape, with four and three institutions respectively). The data suggest that the profession is comprised of younger graduates, and there has been exponential growth over the past two decades. However, the profession is still quite young. By 2000, there were only 136 registered biokineticists, which partly explains the declining numbers of those older than 45 years. Women (67.8%) dominated the profession; however, not to the same extent as HPCSA registered physiotherapists (82.9%).^[17]^ Socio-economic factors were proposed as contributing reasons why physiotherapy is a feminine health care profession.^[20]^ Overall, there were more registered student women than men (57% vs 43%) at Stellenbosch University,^[21]^ suggesting that academic performance also contributed to this trend.

Reasons for the new four-year professional degree includes enhancing the eligibility for the public health sector,^[3]^ and addressing the insufficient experiential training or clinical work-integrated learning in comparison to physiotherapy and occupational therapy.^[22]^ Until recently, biokineticists were exclusively trained at honours (fourth year) level, after completing undergraduate qualifications in Human Movement Studies/Science, Sport Science, Kinesiology or Ergonomics. Honours students register as biokineticists-in-training with the HPCSA and BASA, and complete one of the two years of internship at their respective universities during the honours year. After graduating (and passing the practical clinical assessment), the biokineticists-in-training complete the second year of internship with an HPCSA-accredited training institution or private practice for which they may receive a salary.^[3]^ Completion of the internship and board examination allows for registration with the HPCSA as a biokineticist.

The revised Scope of the Profession and Minimum Standards for Training (MST) for Physiotherapy, Podiatry and Biokinetics (PPB) was amended and approved by the PPB Board in October 2019 with immediate effect.^[8]^ All training programmes should follow the new MST by the next accreditation visit (5-year accreditation cycle). The HPCSA’s mandate is to regulate health professions and to assess if training institutions meet the MST to ensure equitable qualifications. The PPB Board noted that it cannot prescribe how institutions develop their programmes to meet the exit level outcomes. However, the PPB Education, Training and Registrations Committee strongly recommended in 2020 that institutions offering the 3+1-year (Honours) programme migrate to a four-year professional degree.^[22]^ The Minister of Health, following consultation with the HPCSA, intends to Gazette changes to the regulations relating to the Minimum Standards of Education, Training, and Examination in biokinetics, making the four-year professional bachelor’s degree mandatory.^[23]^ Institutions should allow undergraduate pipeline students to complete the academic pathway they embarked on. Universities should inform prospective students of changes to the programme structure and selection criteria timeously.

Seven South African universities offered biokinetics programmes at the Honours level in 2022 and six universities have already migrated to the new four-year degree. One university indicated that they would discontinue their Honours programme in 2023 and will not develop the four-year programme. This means that there will be 12 institutions offering the programme in 2023, six on the old and six on the new programme. Five institutions are migrating to the four-year programme, whilst there is uncertainty about one institution’s plans.

A consequence of the four-year programme is that the current one-year mandatory internship (after completion of the Honours degree) will fall away. Some scholars view this as an advantage of the new pedagogic model and programme structure, since clinical work-integrated learning will commence in the first study year and continue throughout the four-year period.^[3]^ Within the new model, the final clinical assessment will take place just before the students graduate, enabling immediate registration with the HPCSA and entering the workforce. In time, the HPCSA programme accreditation panels would be ideally positioned to comment on the revised four-year curriculum and training and whether it delivers better qualified biokineticists. A community service year for graduates may further strengthen the roll-out of expertise in the public health sector.

A disadvantage of the new model is that private practises may have become reliant on interns, and some may struggle without them. The financial sustainability of academic departments is also cause for concern because the new model may require an overhaul of the existing academic programme. Departments may have to increase their intake of students to remain financially viable since the new programme structure, more teaching hours and supervision of students during clinical rotations, require extra human and financial resources. Unfortunately, some programmes may be discontinued (e.g. the University of KwaZulu-Natal will not offer the biokinetics programme after 2022). In 2021, the BASA President circulated a letter to the Registrars and Heads of Departments of the various institutions. The President urged them to be cognisant of their enrolment numbers because there is pressure on private practices and sites where students complete their clinical training, warning that large graduate cohorts may lead to market saturation.^[24]^

Physical inactivity is the fourth leading risk factor for mortality worldwide.^[25]^ Regular physical activity can address several pathological conditions, thereby reducing population mortality and morbidity rates.^[26]^ A strong body of evidence exists regarding scientifically-based exercise programmes for prevention and management of NCDs and injuries.^[27]^ This open access book chapter summarises empirical evidence on the efficacy of 50 South African biokinetics research publications in tackling the NCD epidemic. Care-based health interventions aimed at improving physical activity are cost-effective in high-risk groups, such as older persons and persons with heart failure.^[28]^ Extensive arguments have been raised for the expansion of biokinetics to the public sector in response to the increased prevalence of NCDs ^[4,14,27,29]^ to alleviate some of the pressure on the public healthcare system by managing and preventing certain chronic, secondary, and associated conditions. Physical activity is an affordable and safe alternative to chronic pharmacological and other medical strategies.^[27]^

Biokinetics was recently included in the Department of Health’s (DOH) National Obesity Strategic Policy.^[30]^ BASA also presented to the DOH regarding inclusion and accreditation within the National Health Insurance (NHI) financing system.^[30]^ Biokineticists could also contribute to reducing re-hospitalisation and over-reliance on the existing healthcare system by providing outpatient exercise rehabilitation services.^[27]^ Home-based physical activity programmes should aim to empower patients and their caregivers, promote quality of life, individual independence, and optimise reintegration into communities.^[27]^

### Recommendations

The current research sets the groundwork for future studies to develop plans for implementation at the tertiary education, private, and public health sector levels to ensure the profession’s long-term sustainability. The profession faces various socio-economic, socio-political, and socio-demographic challenges that should be explored and addressed critically. It is imperative that financial resources and research be strengthened. Health economists should conduct feasibility studies to incorporate biokinetics into the public health sector. Establishing the need for and viability of biokineticists in the public health sector should be prioritised. BASA should lobby the health ministry to introduce state-funded provincial positions, like those for physiotherapy, and occupational therapy. The introduction of community service years may benefit society at large. Clear guidelines should be imposed on how many students the accredited institutions may enrol, train, and deliver, because the new training model may put more pressure on existing private practices, due to the cancellation of the previously mandatory one-year internships. Continuous monitoring and evaluation are needed to avoid market saturation. The profession’s workforce and human resource status (in both the private and public health sectors) should be monitored continuously and strengthened accordingly. The expertise of these healthcare specialists should be utilised to address chronic diseases of lifestyle, disabilities and orthopaedic injuries through clinical exercise therapy and promoting physical activity.

## Conclusion

Based on the period under review (2000 to 2020), the biokinetics profession seems well established, healthy, and steadily growing in South Africa. There are encouraging signs of international expansion. Whilst the demographic profile of registered biokineticists has transformed steadily, the need to transform the profession to become nationally representative is still strong. Women continue to dominate the profession. Regarding training and education, the ongoing migration to four-year professional qualifications is more aligned with the academic requirement of other health professions, like physiotherapy and occupational therapy. There appears to be stability in the number of institutions that offer training and educational programmes, despite the transition to the new four-year pedagogic model and revised programme structure. There is optimism that the proposed NHI financing system would include the profession of biokinetics. Expansion into the public health sector should be the profession’s primary focus.

## Figures and Tables

**Table 1:** Geographical distribution of biokineticists by province in South Africa in 2020

	Province	Number	Percentage of Total	Province Population (2020)	Biokineticists per 100 000 population
**1**	Gauteng	783	43	15 488 137	5.1
**2**	Western Cape	405	22	7 005 741	5.8
**3**	KwaZulu-Natal	185	10	11 531 628	1.6
**4**	Eastern Cape	118	6	6 734 001	1.8
**5**	Free State	101	6	2 928 903	3.4
**6**	Mpumalanga	75	4	4 679 786	1.6
**7**	North-West	64	4	4 108 816	1.6
**8**	Limpopo	54	3	5 852 553	0.9
**9**	Northern Cape	27	2	1 292 786	2.1

	**TOTAL** *	**1812**	**100**	**59 622 351**	**3.0**

* Excluding foreign and unknown categories

**Table 2:** Academic programmes offered for biokineticists in South Africa (2022)

	Province	University	Biokinetics programme (HPCSA registration)			Postgraduate	
			Programme name	Honours degree	4-year degree	Master’s studies	Doctoral studies
**1**	Gauteng	University of Pretoria*	Bachelor of Science Honours in Biokinetics	Yes	No&	Yes	Yes
**2**	Gauteng	Tshwane University of Technology*	Bachelor of Health Sciences in Biokinetics		Yes	Yes	Yes
**3**	Gauteng	University of Johannesburg*	Bachelor of Biokinetics		Yes	Yes	Yes
**4**	Gauteng	University of Witwatersrand#	Bachelor of Health Sciences Honours in Biokinetics	Yes	Unknown	Yes	Yes
**5**	Western Cape	Stellenbosch University*	Bachelor of Science Honours in Biokinetics	Yes	No&	Yes	Yes
**6**	Western Cape	University of Cape Town*	Bachelor of Medical Science Honours in Biokinetics	Yes	No&	Yes	Yes
**7**	Western Cape	University of the Western Cape*	Bachelor of Science Honours in Biokinetics or Bachelor of Arts Honours in Biokinetics	Yes	No&	Yes	Yes
**8**	KwaZulu-Natal	University of KwaZulu-Natal*	Bachelor of Sport Sciences Honours in Biokinetics	Yes+	No	Yes	Yes
**9**	KwaZulu-Natal	University of Zululand*	Bachelor of Science Honours in Biokinetics	Yes	No&	Yes	Yes
**10**	Eastern Cape	Nelson Mandela University*	Bachelor of Health Sciences in Biokinetics		Yes	Yes	Yes
**11**	Free State	University of the Free State*	Bachelor of Biokinetics		Yes	Yes	Yes
**12**	North-West	North-West University*	Bachelor of Health Sciences in Biokinetics		Yes	Yes	Yes
**13**	Limpopo	University of Venda*	Bachelor of Science in Biokinetics		Yes	No	No

* Information verified by Heads of Departments and/or Biokinetics Programme Coordinators.

# Unverified information collated from the University website.

& Universities currently developing/awaiting approval of the 4-year programme. + Last intake of Honours students in 2022 and will not offer the 4-year programme.
